# Valvar bypass surgery to ameliorate persistent lower limb edema caused by post-thrombotic syndrome: a case report and literature review

**DOI:** 10.1186/s13019-024-02601-5

**Published:** 2024-03-21

**Authors:** Yanyang Wang, Liang Zhao, Jie Zhang, Yali Du, Jianfeng Chen, Yingfeng Wu

**Affiliations:** 1 Department of Vascular Surgery, Luhe Hospital, Capital Medical University, No. 82 Xinhua South Road, Tongzhou District, Beijing, 101199 China; 2 Department of Vascular Surgery, Xuanwu Hospital, Capital Medical University, No. 45 Changchun Street, Xicheng District, Beijing, 100053 China

**Keywords:** Chronic venous insufficiency, Post-thrombotic syndrome, Femoral vein, Valvuloplasty, Bypass

## Abstract

Obstruction and/or reflux compromise during venous emptying can facilitate different pathophysiologies in chronic venous insufficiency (CVI). We present a patient with persistent lower limb CVI edema caused by post-thrombotic syndrome (PTS), who responded well to femoral vein valve therapy via axillary vein bypass after unsuccessful valvuloplasty, and led a normal life. During a 12 month observation period, bridging vessels completely restored original anatomical structures. In a literature study, no similar surgeries were reported, but we show that this operation may be feasible in selected patients.

## Introduction

Chronic venous insufficiency (CVI) is a frequent cause of lost working hours and days. In deep veins, obstructions and/or reflux may compromise venous emptying due to different pathophysiologies. CVI is often secondary to deep vein thrombosis (DVT) and is categorized as a secondary etiology, intravenous (ESI) condition in updated Clinical-Etiological-Anatomical-Pathophysiological classifications [1]. If a thrombus lyses or recanalizes, valves may be damaged or destroyed, and deep venous reflux occurs. If the thrombus does not sufficiently lyse or recanalize, the vessel lumen may narrow, becomes occluded, and outflow is obstructed. Peripheral veins may be similarly affected, lose valvular competence, become remodeled, and enlarged with outward flow. Thus, profound venous changes may lead to venous hypertension in superficial veins. This manifestation is called post-thrombotic syndrome (PTS) [2, 3]. When obstruction and reflux are both present, the clinical course becomes more complicated [4].

In this report, we present a patient with stubborn lower limb CVI edema caused by PTS, who successfully responded to axillary vein bypass therapy, and led a normal life.

## Case report

A 48-year-old male was admitted to our hospital with severe CVI of the lower extremity. Fifteen years ago, he developed edema and experienced a feeling of heaviness in both lower limbs, but the right side was worse. He sought medical attention without success (oral drug and unknown treatments). Skin itching and lower pigmentation in both limbs gradually appeared. Three years ago at a local hospital, he was diagnosed with DVT in right lower limb deep veins, including the right external iliac vein and common iliac vein, due to aggravated edema of the right lower extremity and a right foot fracture on the second day after a trauma had occurred. He was implanted with an inferior vena cava filter and the filter was removed in time. Apart from trauma, which is a risk factor for clotting, his doctor at that time did not screen for other clotting factors. After this, he was regularly anticoagulated with warfarin for 1 year, however, lower limb edema had not been alleviated, and right lower limb swelling and heaviness became more serious; moderate pain in the right calf occurred after standing for more than 15 min, which seriously affected his life and work. A systems review revealed a hypertension and hyperlipidemia history. There was no tobacco, alcohol, drug abuse, or recent trauma history, while the family history of arterial and venous thromboembolism was unremarkable. There was no recent history of fever, headaches, visual disturbance, shortness of breath, abdominal pain, nausea, or vomiting.

Physical examination medical records showed no varicose veins in the cross pubic, anterior, and lateral abdominal wall, only superficial varicose veins scattered across both calves. Right lower limb skin color was a mild red color and moderate hemosiderin pigment deposition was observed over the footwear area. Additionally, no evidence of ulceration and healed ulceration was identified. Both lower limbs showed concave edema, which were relatively serious on the right side. The lower leg circumference was 15 cm below the knee, 32.5 cm on the left, and 35 cm on the right. Dorsalis pedis and posterior tibial arteries of the lower limb were palpable. The patient weighed 72 kg. He had normal strength and range of motion in all four limbs. The Villalta scale [5] was 15 points and the Revised-Venous Clinical Severity Score (r-VCSS) [6] was 11 points. Color flow duplex ultrasonography revealed marked full patency of right iliac vein blood flow, right lower extremity venous reflux, with a venous refill time of 5 s and no evidence of obstructed venous outflow. Duplex examinations revealed marked incompetence of the common femoral, superficial femoral, popliteal, and greater saphenous veins. Descending phlebography confirmed incompetence of the superficial femoral vein, with blood flowing back to the lower part of the right calf. Ascending phlebography showed superficial varicosities over the bilateral calves, with no incompetent perforating veins. Based on venous hemodynamic [7], ascending phlebography [8], and descending phlebography evaluations [9], the patient was classified as C4b, EsiAdPro, and Grade IV (Kinster classification; femoral vein reflux below the knee joint level and proximal leg) regurgitation of right femoral vein valve [9], as previously described.

**Fig. 1:**
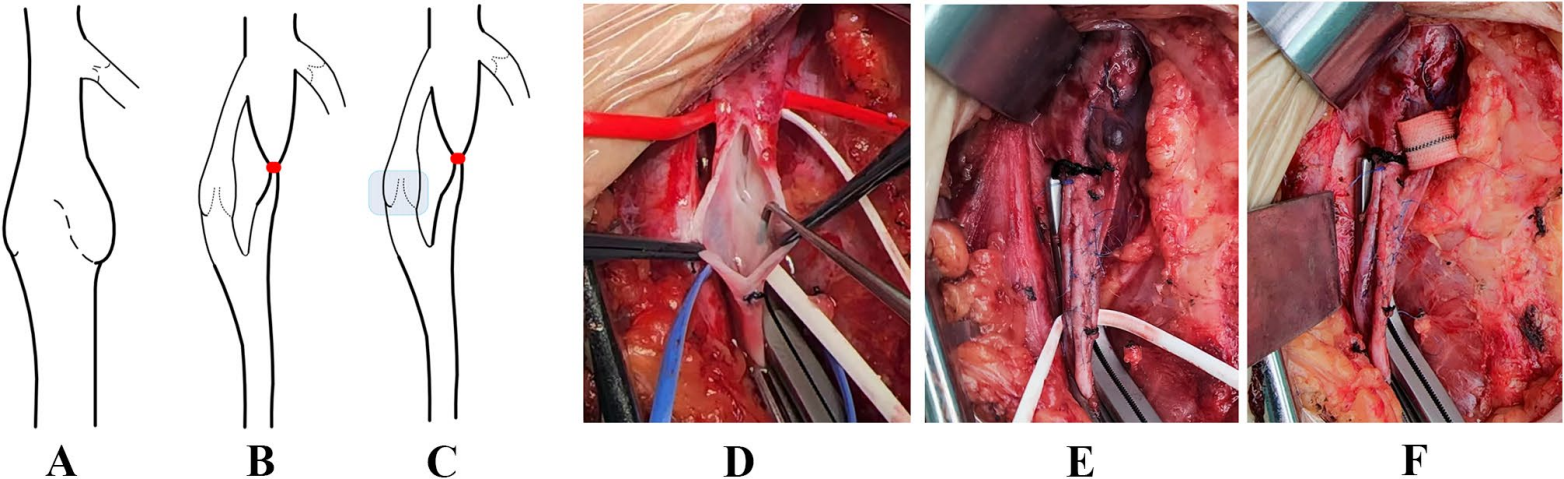
**(A)** The anterior valve of the SFV had almost disappeared, the posterior valve was rigid and thickened. **(B)** The AV was anastomosed with the FV end to side and the SFV ligated. **(C)** The transplanted AV valve segment was covered with the patch. **(D)** The arrow shows the lengthy posterior valve of the first pair of valves of the SFV during surgery. The anterior valve had almost disappeared and could not be shown in images. **(E)** The long arrow refers to the ligated SFV. The short arrow refers to the healthy valve area of the shunt AV, which completely blocked proximal blood flow (strip test). **(F)** The arrow shows the sleeve wrapping (SW) for the AV valve. *Abbrevation * CFV, Common femoral vein; SFV, Superficial femoral vein; GSV, Great saphaneous vein; AV, Axillary vein; SW, Sleeve wrapping

The surgical procedure (described later) was carried out under general anesthesia. After full heparinization, a ‘strip test’ showed that the right femoral vein had severe regurgitation issues. The veins around the proximal end of the superficial femoral vein were blocked. Longitudinal incision of the superficial femoral vein at the first pair of valves, with care taken to protect the internal valve, showed that the anterior valve had almost disappeared and the posterior valve was rigid and thickened. Considering that this flap could not be normally repaired, we performed axillary vein valve transplantation. An anterograde incision was made at the projection of the body surface of the mid and distal axillary vein, with valves marked by ultrasound before surgery. We identified a transplantable axillary valved vein of 5 mm in diameter. A strip test showed that the function of the axillary vein valve, prepared for transplantation, was normal. In order to match the transplant site, we trimmed the axillary vein segment with valves for transplantation, which was approximately 4 cm long.

The axillary vein was smoothly removed and anastomosed with the femoral vein end to side at the appropriate position of the proximal and distal part of the first pair of diseased valves of the superficial femoral vein. After proximal anastomosis, we opened the proximal block to confirm that the tube wall at the valve graft filled without reflux. We ligated the superficial femoral vein segment corresponding to the bridging vessel. We used an 8 × 75 mm Maquet patch to sleeve-wrap the transplanted axillary vein valve segment [8]. A strip test was repeated after valvuloplasty to confirm no reflux. Indwelling drainage in inguinal and axillary incisions were then sutured and the operation was completed (Fig. 1).

**Fig. 2:**
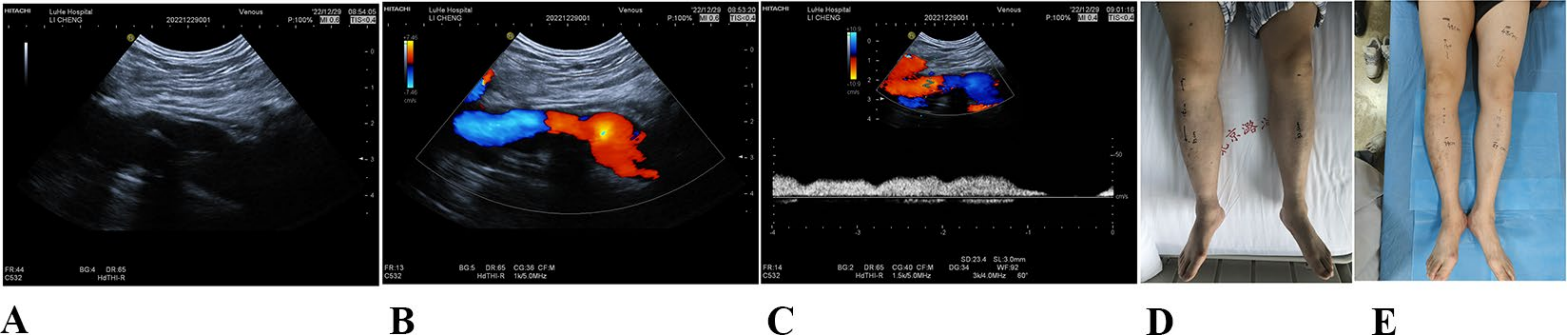
Ultrasound at 3 months after surgery **(A)** The long solid arrow shows the SFV, and the long arrow shows the AV. The short solid arrow shows the ring, and the cross mark shows the ligation point of the SFV. **(B)** The diverted axillary vein is unobstructed; the arrow shows the healthy axillary vein valve. **(C)** During the valsalva maneuver, no blood reflux was recorded in the diverted vein valve. **(D** and **E)** The patients lower limbs before and at 3 months after surgery, which showed slight edema in the right calf, but the leg circumference at 15 cm below the knee was 1 cm less than that before surgery

Heparinization was provided for 3 consecutive days after surgery, maintaining Activated Partial Thromboplastin Time = 1.5–2.5 at all times. Then, the patient received adequate anticoagulation therapy (rivaroxaban for the first three weeks: 15 mg, bid; After 3 weeks–3 months: 20 mg, Qd for 3 months). Edema, heaviness, and pain in the right lower limb were significantly relieved after surgery. The patient continued to maintain pressure treatment from medical elastic stockings. The patient was rechecked at 1 and 3 months after surgery and showed no obvious regurgitation of the right femoral graft vein valve. Valve function and d-dimer values were normal and no new thrombosis was identified. The patient returned to normal life and work. At 3 months post-surgery, right lower leg pigmentation became lighter, tension was reduced in varicose veins in the lower leg, and the right ankle was slightly edematous (Fig. 2). Heavy feelings, pain, and other lower limb symptoms disappeared, and edema was well controlled by elastic stockings. The Villalta scale reduced to 5 points and the r-VCSS was reduced to 5 points. Ultrasound (Fig. 2) showed no thrombosis near the original ligated superficial femoral vein, the valve in the transplanted bypass vessel opened and closed well, and no reflux was observed when the valsalva maneuver was performed. The patient was very satisfied. When he revisited at 3 months after surgery, we advised him to discontinue anticoagulation and continue with long-term elastic compression stockings. It has been 1 year since the surgery, and the patient has been wearing elastic compression stockings. Lower limb swelling symptoms have all disappeared. A follow-up ultrasound (local hospital) showed that the bridging blood vessel was unobstructed and its valve showed no reflux. We obtained the patients consent for this publication.

## Discussion

CVI pathophysiology is represented by events that occur within larger superficial and deep veins and events that subsequently occur in the microcirculation and surrounding skin tissue [10]. PTS is a set of CVI symptoms and signs caused by impaired venous outflow due to deep venous obstruction and/or reflux following a DVT [11]. Typical signs are pain with calf compression, varicose veins, edema, and skin color change, like Venous Leg Ulcers (VLUs) [4]. This syndrome occurs in 20–50% of DVT patients, maybe more, of whom 5–10% develop severe PTS, such as VLU. Our patient had CVI symptoms before DVT. Therefore, we considered that CVI disease before DVT was one of the DVT causes on the patient’s right side. Three years after right lower extremity DVT, the patient had obvious lower limb edema, heaviness, pain, varicose veins, and pigmentation before surgery, which was completely consistent with a PTS diagnosis. The Villalta scale was 15 points before surgery which indicated severe PTS.

In patients with PTS, conservative treatment is the first option and consists of supervised exercise training, compression treatment (usually with elastic compression stockings), and pharmacotherapy, including venoactive drugs (e.g., horse chestnut extract) [12]. The patient had tried all conservative treatment in the previous 2 years, with no positive outcomes, thus life and work were seriously affected. Therefore, urgent effective treatments were required.

Surgically treating deep vein incompetence (DVI) is required if patients without outflow obstruction or previously corrected outflow obstruction, and conservative DVI lower limb management has failed, and also severe chronic venous disease (CVD) symptoms and signs persist. Only selected patients with axial reflux at thigh level, across the popliteal vein, and into calf veins are considered for intervention [13] with endovenous or surgical techniques [14]. An ultrasound examination of our patient showed that the thrombus had disappeared completely. An angiography also showed that the right femoral vein flowed back to the distal part of the calf veins, consistent with surgical intervention indications.

In our patient, angiography before surgery and a non-existent anterior valve and rigid posterior valve during surgery indicated that valvuloplasty or femoral vein transposition was not advisable. Other reconstruction options included the transplantation of a vein segment with a competent valve (usually the axillary vein) or neovalve creation from a thickened vein wall or an artificial prosthetic valve [15]. Overall, after open valve reconstruction for DVI, ulcer-free rates vary between 54% and 100% for up to 5 years [16]. But this may be attributed to superficial reflux treatment or compression therapy in some patients. As no comparative studies were available, it is impossible to recommend different types of surgery for DVI [16]. Unfortunately, neovalve or artificial prosthetic valves have not yet come to operating rooms, although some neovalve cases are presented, the only choice is valve transplantation [17, 18]. In axillary vein valve transplantation, in our case, the tube diameter of the proximal and distal valved axillary vein did not coincide with the femoral vein, so we performed end to side instead of anterior end-to-end anastomosis. More importantly, anastomosis was performed to avoid axial reflux, which may have caused short term dysfunction in vulnerable axillary vein valves. Danielsson et al. scanned the legs of 83 patients with active VLUs, and axial reflux was detected in 79% [19]. Surgically eliminating major venous obstruction and axial reflux to the venous ulcer bed, and providing adequate compression therapy, are the two major treatment modalities used for venous ulcer healing [20]. Inspired by this, we speculate that this method of end to side may reduce axial reflux and achieve long-term bypass vein patency during lower limb vein surgery. Currently, no research has been performed in this area, therefore more in-depth research is required.

To avoid femoral vein blood flow distortions and target vessel occlusion, we ligated the femoral vein truck. Also, based on our experience, we performed a sleeve wrapping operation on the graft valve area of the axillary vein, with a view to achieving good long-term graft valve function [21].

Surgeries correcting deep vein reflux are challenging. From our literature review, no similar operations were reported. During 1 year of observation of our patient, the bridging vessel completely restored original anatomical blood flow. This successful case history shows that this surgery may be feasible for selected patients.

## Data Availability

Not applicable.
